# SOX2 Regulates Growth, Expression of Basal/Luminal Markers, and Chemotherapy Response in Urothelial Carcinoma

**DOI:** 10.3390/cells14130949

**Published:** 2025-06-20

**Authors:** Nelofar Nargis, Abigail Lind, Adam Sczepanski, Randi Herndon, Olivia Smiley, Seema Somji, Donald A. Sens, Aaron A. Mehus

**Affiliations:** Department of Pathology, School of Medicine and Health Sciences, University of North Dakota, Grand Forks, ND 58202, USA; nelofar.nargis@und.edu (N.N.); abby3148@live.com (A.L.); adam.sczepanski@und.edu (A.S.); randi.herndon@und.edu (R.H.); olivia.smiley@und.edu (O.S.); seema.somji@und.edu (S.S.); donald.sens@med.und.edu (D.A.S.)

**Keywords:** SOX2, basal, urothelial carcinoma, arsenite, cisplatin, squamous differentiation

## Abstract

Urothelial carcinoma (UC) is a common genitourinary malignancy. Smoking, exposure to arsenic in drinking water, and age can increase the risk of developing UC. Neoadjuvant cisplatin-based chemotherapy prior to radical cystectomy is the standard treatment for the muscle invasive form of UC (MIUC). Tumors of the basal/squamous (Ba/Sq) subtype of MIUC are aggressive, express basal keratins (KRT5, 6, and 14), are associated with squamous differentiation (SD), and frequently develop chemotherapy resistance. The SOX2 transcription factor is a marker of UC stem cells, and its expression is associated with poor overall and disease-free survival. We hypothesized that the attenuation of SOX2 would reduce the expression of basal keratins and increase the chemotherapy response in human UC cells. For this study, we performed lentiviral knockdown (KD) of SOX2 expression in two separate arsenite (As^3+^)-transformed UROtsa (As_I, As_II), 5637, and RT4 cells. Cellular growth and colony-forming ability was inhibited in all UC cell lines after SOX2 KD. We demonstrate that SOX2 KD in the UC cells of the Ba/Sq subtype (As_I, As_II, 5637) decreased the expression of stem-associated proteins, oncoproteins, and basal keratins. Additionally, there was an induction of several luminal markers and enhanced cisplatin sensitivity following the repression of SOX2. Lastly, proteomics revealed reductions in lipid-, cholesterol-, and interferon-signaling pathways after SOX2 KD. This study provides a better understanding of the regulation of key genes responsible for defining the Ba/Sq subtype of UC and demonstrates that the inhibition of SOX2 improves chemotherapy response in UC.

## 1. Introduction

In 2025, it is estimated that there will be 84,870 new cases of bladder cancer diagnosed within the United States, and three-fourths of those cases will be in men [1]. Urothelial carcinoma (UC) is responsible for most bladder cancer cases. Roughly 75% of UCs are highly recurrent and characterized as non-muscle invasive (NMIUC), where the cancer is in the transitional epithelium and has not spread into the deeper layers of the bladder wall. However, the remaining 25% of UCs are diagnosed as being muscle invasive (MIUC), where the cancer has grown into the muscle wall and the prognosis is worse [2]. MIUC tumors can further be classified as being of the luminal or basal/squamous (Ba/Sq) subtypes. Tumors of the luminal subtype usually represent less severe cases and are often characterized by the expression of the transcription factors PPARG, FOXA1, and GATA3. The Ba/Sq subtype is aggressive, has poor survival, and is often associated with squamous differentiation (SD). The distinguishing feature of the Ba/Sq subtype is the elevated expression of the basal keratins (KRT5, KRT6, and KRT14) and P63 [3,4]. Patients that have MIUC tumors with SD frequently develop chemoresistance, have higher rates of nodal metastasis, and have significantly lower overall survival (OS) and recurrence-free survival (RFS) [5,6,7].

Our lab has transformed normal human urothelial (UROtsa) cells into malignant cells by environmentally relevant exposure to arsenite (As^3+^) [8]. The tumors generated from these cells display focal areas of SD, and their gene expression profiles have been correlated to the basal subtype of MIUC [9]. Our studies have shown that these As^3+^-transformed cell lines contain cancer stem cells (CSCs) that can grow as urospheres that are enriched with genes associated with SD [9,10,11]. These As^3+^-transformed UROtsa cells overexpress transcription factor SOX2, which has been shown to drive malignant stemness and proliferation in UC cells [11,12,13]. We have previously demonstrated that treatment with pevonedistat (PVD), a neddylation inhibitor and potent SOX2 inhibitor, reduces cell growth, attenuates urosphere formation, and induces apoptosis within these As^3+^-transformed cells, while having minimal effects on the non-transformed parent UROtsa cells [11].

SOX2 is one of the original Yamanaka factors (OCT4, SOX2, KLF4, and c-MYC), wherein the ectopic expression of these factors together can convert terminally differentiated somatic cells into induced pluripotent stem cells (iPS) [14]. SOX2 expression is absent in normal human and mouse urothelial cells but is expressed in UC tumors [15]. It is reported that SOX2 is focally expressed in bladder tumors; therefore, its expression can be underestimated but is generally positive in 40–43% of UC tumors [16,17]. SOX2 expression is higher in muscle-invasive compared with non-invasive papillary UC and its expression correlates with advanced pathological grade and reduced overall survival and recurrence-free survival in UCs [13,16,18]. In UC, SOX2 expression is suggested to be a marker for a subpopulation of cancer stem cell (CSCs) that co-express keratin 14 (KRT14) and CD44v6, and these CSCs are responsible for the maintenance, invasion, and progression of UC tumors [15,18]. SOX2 is known to play a role in cisplatin resistance in other tumor types [19,20,21,22]. However, given the aggressive nature and frequency of MIUC tumors that become resistant to cisplatin-based treatment, very little is known about the role that SOX2 plays in regulating the Ba/Sq subtype or chemotherapy response in UC.

Proteomics in cancer research can be utilized to identify specific proteins, protein modifications, or biological pathways related to cancer development, progression, and/or therapy responses. Protein knockdown (KD) studies in combination with proteomics is a powerful tool that can be used to evaluate the influence that one specific protein has on the global expression of other proteins within cancer cells. The information from these studies can be used to target specific pathways, which can be used for developing a personalized approach to chemo- or immunotherapies.

The primary objective of this study was to evaluate whether there are UC subtype-specific effects in attenuating SOX2 expression in the cells of either the basal (UROtsa As_I, As_II, and 5637 cells) or luminal (RT4 cells) UC subtypes. Specifically, we were interested in understanding the regulatory role of SOX2 on growth, basal/squamous expression, chemosensitivity, and identifying SOX2-dependent biological pathways in UC.

## 2. Results

### 2.1. Stable SOX2 Knockdown in UC Cells

**Figure 1:**
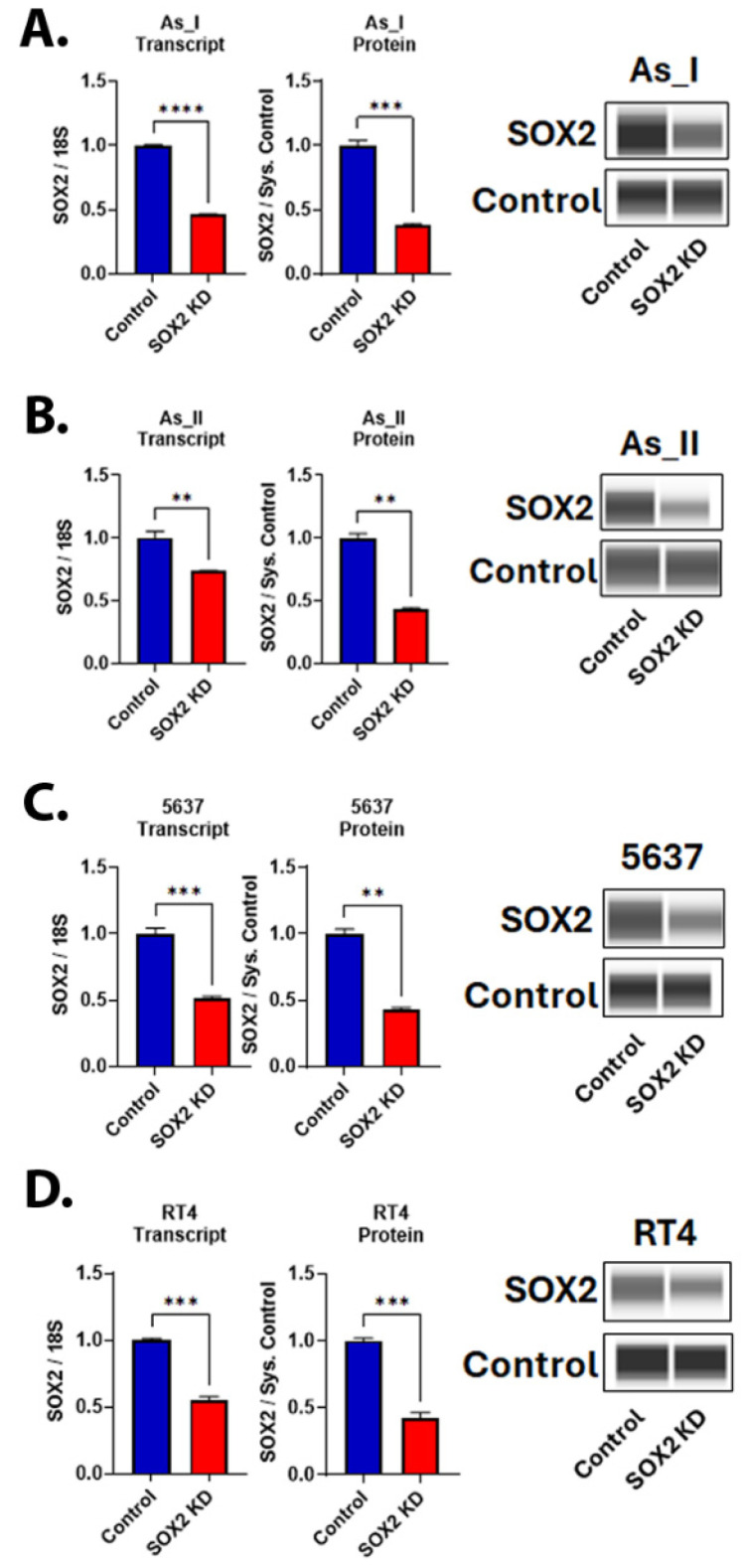
Knockdown of SOX2 in UC cells. (**A**) SOX2 gene and protein expression after SOX2 knockdown (KD) in (**A**) UROtsa As_I cells, (**B**) UROtsa As_II cells, (**C**) 5637 cells, and (**D**) RT4 cells. All data are plotted as fold-change compared to the scramble control for each cell line. Gene expression was normalized to the 18S housekeeping gene, and protein expression was normalized to the Jess system control. Gene data represent *n* = 3 and protein data represent n = 2. The values reported are mean ± SEM. A *t*-test was performed, and asterisks indicate significant differences from the control (** *p* < 0.01, *** *p* < 0.001, **** *p* < 0.0001). KD: knockdown; SOX2: transcription factor SOX2.

We first performed lentiviral transduction in biological triplicates (a,b,c) to evaluate the knockdown (KD) efficiencies of two separate shRNA sequences (shRNA #1 and #2) that target human SOX2 or a scramble (control) shRNA within three separate UC cell lines (UROtsa As_I, 5637, and RT4). Figure S1 displays the resulting SOX2 protein levels in each cell line after using each shRNA sequence alone or in combination with each other (SOX2 shRNA #1&2). The results demonstrate that shRNA #1 alone had variable effects, showing ~25–75% knockdown of SOX2 in As_I and 5637 cells, respectively. However, shRNA #1 was not effective in reducing SOX2 within the RT4 cells. shRNA #2 provided the most consistent KD of SOX2 in all three cell lines, reducing the protein at least 50–75% in all UC cell lines. An additive reduction using the combination of both shRNA sequences was only seen for the 5637 cell line. For consistency, we used shRNA #2 for the remainder of the experiments in this manuscript. Figure 1 shows that both the transcript and protein levels of SOX2 were attenuated after SOX2 KD in all four UC cell lines (As_I, As_II, 5637, and RT4). All uncropped blots for this manuscript are provided in Supplementary Figures S2, S4, S6–S8. All remaining gene expression data for this manuscript are provided in Supplementary Figure S5.

### 2.2. Morphology and Growth After SOX2 Knockdown in UC Cells

**Figure 2:**
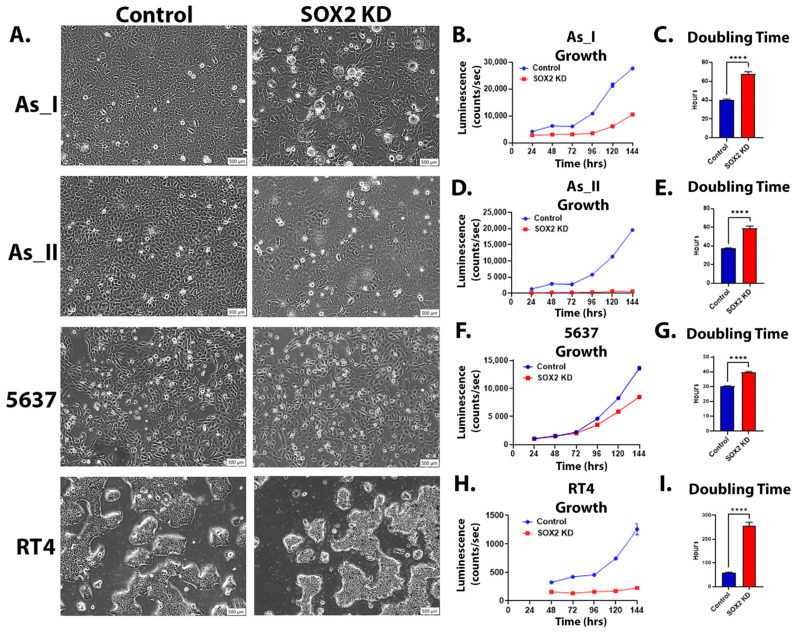
Morphology and growth after SOX2 knockdown in UC cells. (**A**) Brightfield microscope images (10× magnification, scale bar = 500 µm) of UC cells after SOX2 knockdown. (**B**,**D**,**F**,**H**) Luminescence intensity plotted over 24, 48, 72, 96, 120, and 144 h for control and SOX2-knockdown As_I, As_II, 5637, and RT4 cells. A total of 2500 cells were initially seeded for growth and measured according to their luminescence on a 96-well plate. (**C**,**E**,**G**,**I**) Calculated doubling times obtained from the luminescence intensity curve. Luminescence growth represents *n* = 8 and the mean intensity ± SEM is plotted. A *t*-test was performed, and asterisks indicate significant differences from the control (**** *p* < 0.0001). KD: knockdown; SOX2: transcription factor SOX2.

We next evaluated the cellular morphology of the UC cells following KD of SOX2 levels. We observed that the As_I and As_II cells were discernably enlarged while the 5637 cells were somewhat enlarged, and the RT4 cells appeared to be similar in size compared to the control cells after SOX2 KD (Figure 2A). Although the SOX2 KD RT4 cells were not enlarged compared to the control cells, they did appear unhealthy/stressed, and we noticed that they were growing more slowly compared to the control cells. In fact, we noticed that SOX2 KD for all the UC cell lines resulted in more slowly growing cells; therefore, we next wanted to measure the cellular growth rates for all the UC cell lines following SOX2 KD. Since our lentiviral vectors express a luciferase reporter, we wanted to utilize this characteristic (described previously) to monitor the growth/viability of the UC cells over time [23]. We first began by serially diluting and seeding cells to generate a luminescence kinetic curve. This is performed to determine the optimal time point after substrate (luciferin) addition to measure luminescence (when the signal stabilizes). As shown in Figure S3A, 10 min after substrate addition is the optimal time to measure the luminescence signal for all the UC cell lines. Therefore, this was the time point that was used to monitor growth daily. To verify the accuracy of the assay and ensure that cell number correlates to luminescence intensity, we performed a serial dilution and performed the assay. Figure S3B shows that there is a strong correlation (R^2^ < 0.99) between the luminescence intensity and the number of cells seeded for all cell lines. We then assessed cellular growth over six days between the control and SOX2 KD UC cells (Figure 2B,D,F,H). The results demonstrate that the SOX2 KD cells grow more slowly than the control cells, which is also reflected in elevated doubling times (Figure 2C,E,G,I).

### 2.3. Effect of SOX2 Knockdown on Colony Formation in UC Cells

**Figure 3:**
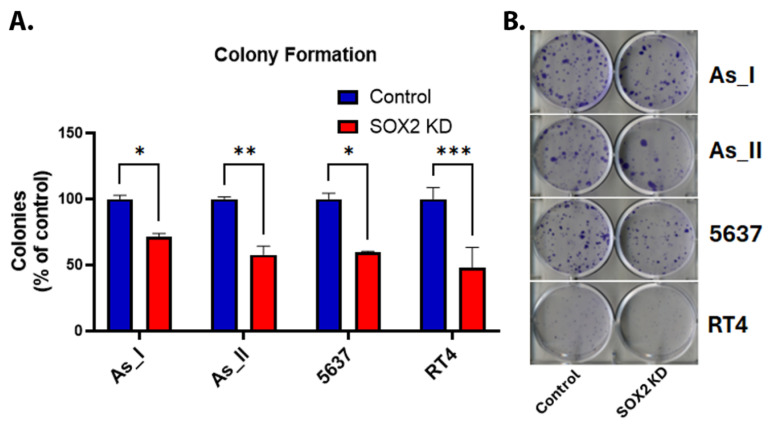
Effect of SOX2 knockdown on colony formation in UC cells. (**A**) Quantification of colonies (expressed as percentage of control). (**B**) Image of crystal-violet-stained colonies in the UC cells. Initially, 200 cells/well were seeded in a 6-well plate and cultured for 14 days before staining the resulting colonies. The colony counts for control and SOX2-knockdown cells for each cell line were performed with *n* = 3. The values reported are mean ± SEM. A *t*-test was performed, and asterisks indicate significant differences from the control (* *p* < 0.05, ** *p* < 0.01, *** *p* < 0.001). KD: knockdown; SOX2: transcription factor SOX2.

SOX2 is considered an important stem marker for UC cells, and in vitro colony formation is reflective of the stem-like nature and reproductive viability of cells. Therefore, we assessed colony-forming abilities between the control and SOX2 KD UC cells [15,24,25]. Figure 3A,B shows that colony-forming abilities were impaired following SOX2 KD in all UC cell lines. On average, there was a ~50% reduction in colonies in the SOX2 KD cells compared to the control cells.

### 2.4. SOX2 Regulates the Expression of Stem-Associated Proteins and Oncoproteins in UC Cells

**Figure 4:**
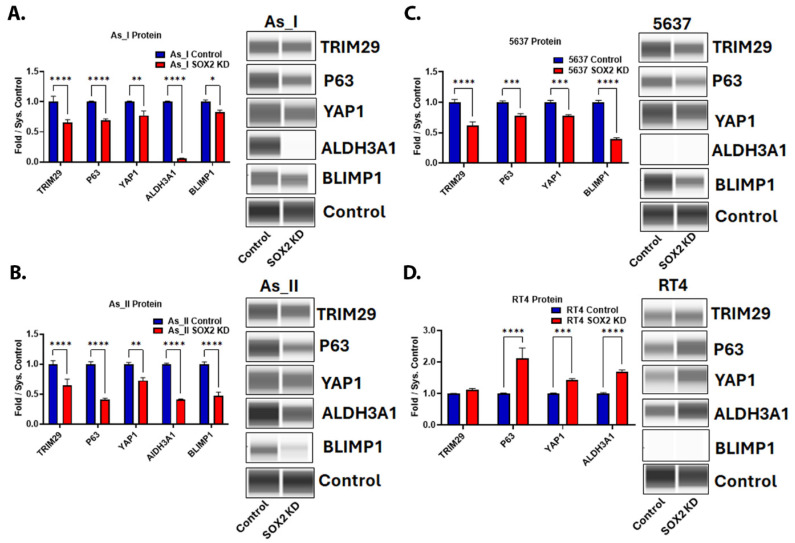
SOX2 regulates the expression of stem-associated proteins and oncoproteins in UC cells. Quantification and Western blot images of proteins in (**A**) UROtsa As_I cells, (**B**) UROtsa As_II cells, (**C**) 5637 cells, and (**D**) RT4 cells after SOX2 knockdown. All data are plotted as fold-change compared to the scramble control for each cell line. Protein expression was normalized to the Jess system control. Protein data represent *n* = 2. The values reported are mean ± SEM. A *t*-test was performed, and asterisks indicate significant differences from the control (* *p* < 0.05, ** *p* < 0.01, *** *p* < 0.001, **** *p* < 0.0001). KD: knockdown; SOX2: transcription factor SOX2; TRIM29: tripartite motif-containing protein 29; P63: tumor protein 63; YAP1: transcriptional coactivator YAP1; ALDH3A1: aldehyde dehydrogenase, dimeric NADP-preferring; BLIMP1: PR domain zinc finger protein 1.

Since SOX2 is a key regulator of UC stemness, we also wanted to measure the levels of several proteins associated with stem-like properties/maintenance and oncoproteins. TRIM29 is an oncoprotein and a major driver of tumor formation/invasion in UC [26]. The P63 transcription factor is activated in basal MIUC, where it is said to regulate the expression of TRIM29 and the basal UC gene program [3,26,27,28]. YAP1 signaling has roles in UC stemness and cisplatin resistance [29,30,31]. ALDH3A1 plays roles in the maintenance of cancer stem cells and cisplatin resistance [32,33]. BLIMP1 plays critical roles in the terminal differentiation of epithelial cells, and its transcript (PRDM1) was found to be enriched within areas of SD in UC tumors [34,35]. The repression of SOX2 in the UC cells of the basal/squamous (Ba/Sq) subtype of MIUC (As_I, As_II, and 5637) resulted in a decreased protein expression of TRIM29, P63, YAP1, and BLIMP1 (Figure 4A–D). ALDH3A1 protein was decreased after SOX2 KD in the As_I and As_II cells, but the protein was not expressed at detectable levels in 5637 cells (Figure 4C); however, the transcript was expressed and was reduced after attenuating SOX2 expression. Interestingly, in the RT4 cells, which are a model of luminal, papillary non-muscle invasive UC (NMIUC), SOX2 KD had opposing effects. The protein levels of P63, YAP1, and ALDH3A1 increased after SOX2 KD. Also, BLIMP1 protein was only detected in the cells of Ba/Sq subtype of MIUC and not the luminal subtype of NMIUC cells. Taken together, these results indicate there are some subtype-dependent differences after SOX2 depletion and that TRIM29, P63, YAP1, ALDH3A1, or BLIMP1 are not solely responsible for the reduced colony-forming abilities we observed, which is dependent on SOX2 directly and/or some other downstream target(s) of SOX2.

### 2.5. Immunohistochemical Staining of YAP1, ALDH3A1, and BLIMP1 in Tumors Derived from RT4 and UROtsa As_I UC Cells

**Figure 5:**
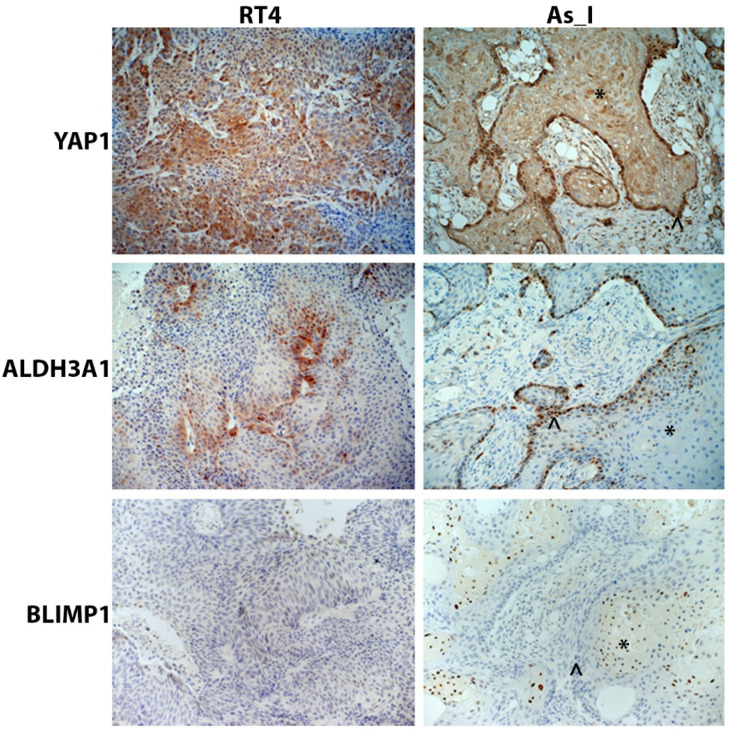
Immunohistochemical staining of YAP1, ALDH3A1, and BLIMP1 in tumors derived from RT4 and UROtsa As_I UC cells. YAP1 displayed mainly cytoplasmic staining and some nuclear expression in both RT4 and As_I tumors. ALDH3A1 displayed mainly cytosolic staining in RT4 tumors but mostly nuclear staining As_I tumors. BLIMP1 expression was negative in RT4 tumors and had nuclear expression in As_I tumors. The asterisks (*) indicate well-differentiated/squamous areas, and the caret (^) indicates basal areas within the As_I tumors. The brown color indicates the presence of the protein, whereas the blue/purple color indicates the nuclei that are stained with the counterstain hematoxylin. All images are at a magnification of 20×. Data represent *n* = 5/group. YAP1: transcriptional coactivator YAP1; ALDH3A1: aldehyde dehydrogenase, dimeric NADP-preferring; BLIMP1: PR domain zinc finger protein 1.

Since YAP1, ALDH3A1, and BLIMP1 were found to be regulated by SOX2 in the Ba/Sq subtype of MIUC cells, we wanted to confirm their expression/localization in vivo from tumors derived from As_I cells and RT4 cells (for comparison) using immunohistochemical (IHC) staining. We have previously reported low to no staining of P63 and TRIM29 in tumors derived from RT4 cells and positive staining for the proteins in tumors derived from As_I cells [36]. Additionally, we have reported the positive nuclear expression of SOX2 in the well-differentiated squamous areas and also the non-differentiated basal areas of tumors derived from As_I cells [11]. YAP1 was expressed in both As_I and RT4 tumors and displayed mostly cytoplasmic staining and some nuclear expression in both tumor types (Figure 5). Within tumors derived from the As_I, YAP1 was expressed in both the squamous and basal areas of the tumors. ALDH3A1 demonstrated positive staining in both As_I and RT4 tumors and displayed mainly cytoplasmic staining in RT4 tumors and some cytoplasmic but mostly nuclear staining in As_I tumors. Within As_I tumors, ALDH3A1 expression was confined mostly to the basal areas of the tumors. As expected, BLIMP1 was not expressed in tumors derived from the RT4 cells, which agrees with our in vitro results. BLIMP1 displayed positive nuclear expression in tumors derived from the As_I cells, and its expression was limited to the squamous areas of tumors, which agrees with transcript detection of PRDM1 in human UC tumors [35].

### 2.6. Expression of Basal Keratins After SOX2 Knockdown in UC Cells

**Figure 6:**
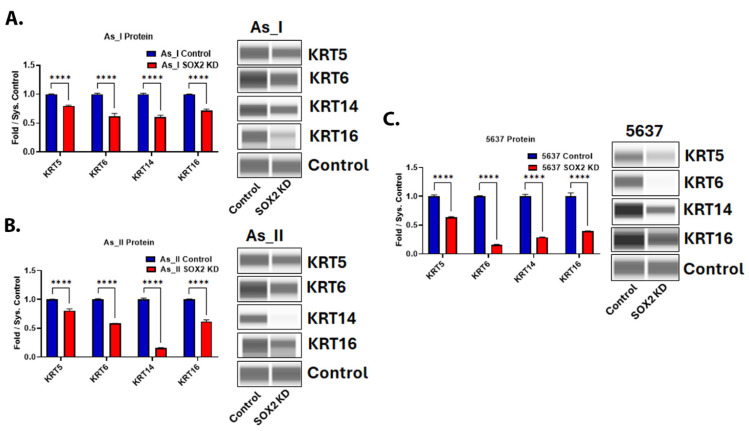
Expression of basal keratins after SOX2 knockdown in UC cells. Quantification and Western blot images of proteins in (**A**) UROtsa As_I cells, (**B**) UROtsa As_II cells, and (**C**) 5637 cells after SOX2 knockdown. All data are plotted as fold-change compared to the scramble control for each cell line. Protein expression was normalized to the Jess system control. Protein data represent *n* = 2. The values reported are mean ± SEM. A *t*-test was performed and asterisks indicate significant differences from the control (**** *p* < 0.0001). KD: knockdown; SOX2: transcription factor SOX2; KRT5: keratin, type II cytoskeletal 5; KRT6: keratin, type II cytoskeletal 6; KRT14: keratin, type I cytoskeletal 14; KRT16: keratin, type I cytoskeletal 16.

Since SOX2 expression is higher in MIUC vs. NMIUC cases and the Ba/Sq subtype of MIUC is characterized by the expression of the basal keratins (KRT5, 6, 14, and 16), we wanted to evaluate whether SOX2 was responsible for regulating their expression. In all three cell lines of the Ba/Sq subtype, SOX2 KD resulted in a decreased protein expression of the basal keratins (Figure 6A–C). As expected, these protein markers of the Ba/Sq subtype of MIUC were not detected in the RT4 cell line (Figure S6), and their transcript levels were very low.

### 2.7. Expression of Proteins Associated with the Luminal Subtype of UC After SOX2 Knockdown

**Figure 7:**
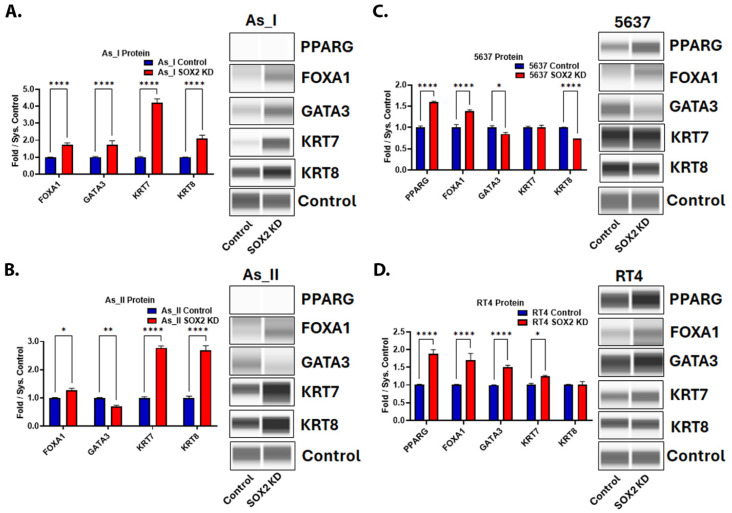
Expression of proteins associated with the luminal subtype of UC after SOX2 knockdown. Quantification and Western blot images of proteins in (**A**) UROtsa As_I cells, (**B**) UROtsa As_II cells, (**C**) 5637, and (**D**) RT4 cells after SOX2 knockdown. All data are plotted as fold-change compared to the scramble control for each cell line. Protein expression was normalized to the Jess system control. Protein data represent *n* = 2. The values reported are mean ± SEM. A *t*-test was performed and asterisks indicate significant differences from the control (* *p* < 0.05, ** *p* < 0.01, **** *p* < 0.0001). KD: knockdown; SOX2: transcription factor SOX2; PPARG: peroxisome proliferator-activated receptor gamma; FOXA1: hepatocyte nuclear factor 3-alpha; GATA3: trans-acting T-cell-specific transcription factor GATA-3; KRT7: keratin, type II cytoskeletal 7; KRT8: keratin, type II cytoskeletal 8.

Since we demonstrated that SOX2 KD decreased basal keratin expression, we wanted to evaluate the effects of SOX2 depletion on the expression of some of the luminal markers of UC. Figure 7A–D demonstrates that SOX2 KD increased the expression of FOXA1 in all the UC cell lines. Keratin 7 (KRT7) was also elevated after SOX2 KD in three cell lines (As_I, As_II, and RT4). GATA3 increased in As_I and RT4 cells but decreased in As_II and 5637 cells after SOX2 KD. PPARG was not detected in the As_I or As_II cells but was increased from SOX2 KD in the 5637 and RT4 cells. Keratin 8 (KRT8) increased in As_I and As_II cells, was unchanged in the RT4 cells, and decreased in the 5637 cells after SOX2 KD. To summarize, FOXA1 and KRT7 were the most consistently induced luminal markers following SOX2 KD, which suggests that SOX2 may be repressing their expression.

### 2.8. SOX2 Regulates Chemotherapy Response in UC Cells

**Figure 8:**
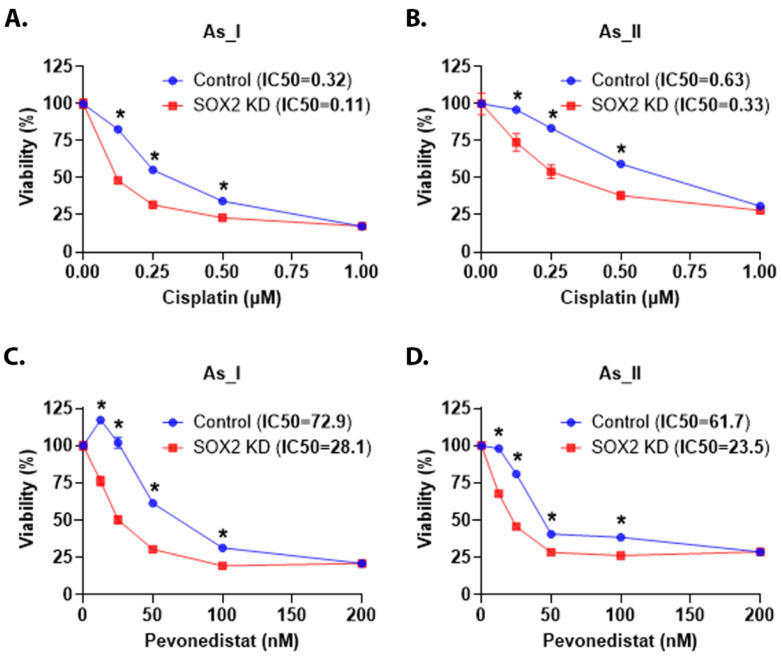
SOX2 regulates chemotherapy response in UC cells. (**A**,**B**) Viability (expressed in percentage) after 0.00 (control), 0.13, 0.25, 0.50, and 1 µM cisplatin treatment for 72 h in the UROtsa As_I and As_II cells, respectively. (**C**,**D**) Viability after 0.00 (control), 12.5, 25.0, 50.0, 100.0, and 200.0 nM pevonedistat treatment for 72 h in the UROtsa As_I and As_II cells, respectively. The IC50 values are reported in the top panel (next to sample names). Blue bars indicate control viability while red bars indicate viability in the SOX2 KD cells. In total, 2500 cells were seeded/well in a 96-well plate, and there were eight replicates (*n* = 8) for each dose. Twenty-four hours after seeding, the cells were dosed with either cisplatin or pevonedistat for 72 h. The reported values are mean ± SEM. A *t*-test was performed, and asterisks indicate significant differences from the control (* *p* < 0.05). KD: knockdown; SOX2: transcription factor SOX2; %: percentage.

Patients with MIUC are routinely treated with cisplatin-based neoadjuvant chemotherapy and frequently acquire chemoresistance. It has also been reported that patients with UC tumors with SD are less responsive to cisplatin-based chemotherapies [37,38]. The As_I and As_II cells are a model of the basal subtype of MIUC capable of producing tumors with SD [39,40]. Therefore, we wanted to determine whether KD of SOX2 would increase the cisplatin sensitivity of these cells. We monitored viability after cisplatin treatment (Figure 8A,B). The results demonstrate that the SOX2-knockdown cells had lower IC50 values compared to the scramble control cells, which indicates increased sensitivity to cisplatin. We have previously reported that pevonedistat (PVD) enhances the efficacy of cisplatin treatment in UC cells [11]. Therefore, we were interested to see if depleting the SOX2 levels in the As_I and As_II cells would also increase their sensitivity to PVD. Figure 8C,D shows that the SOX2 KD cells had lower IC50s and are more sensitive to PVD. Taken together, these results suggest that reducing levels of SOX2 in UC cells of the Ba/Sq subtype of MIUC leads to increased chemotherapy response to cisplatin and PVD treatments.

### 2.9. Proteomics Identifies That Lipid/Cholesterol- and Interferon-Signaling Pathways Are Downregulated After SOX2 KD

**Figure 9:**
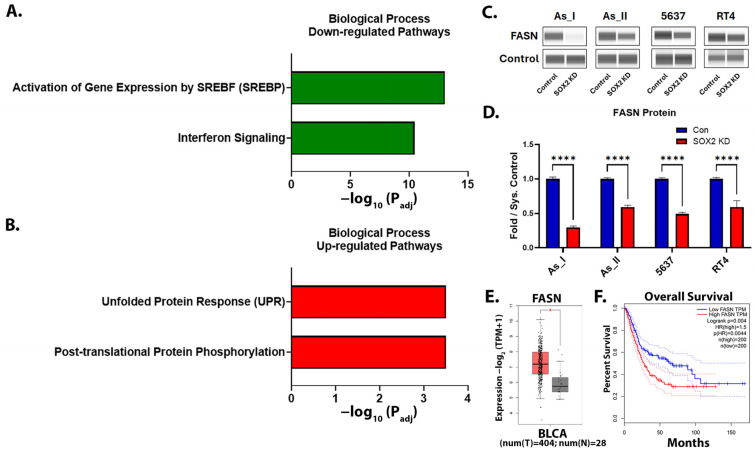
Proteomics identifies that lipid/cholesterol- and interferon-signaling pathways are downregulated after SOX2 knockdown¯. (**A**) The top two biological process pathways downregulated or (**B**) upregulated after SOX2 KD in UROtsa As_II cells. (**C**) Western blot images and (**D**) quantification of FASN protein levels after SOX2 KD in UC cells. (**E**) The GEPIA2 boxplot of FASN transcript expression in normal and BLCA tissues. Red bars represent tumor (T) levels and grey bars represent normal (N) tissue levels. (**F**) The GEPIA2 survival curve for FASN. The blue dashed lines indicate the 95% confidence interval (CI) for the low FASN expression group and the red dashed lines indicate the 95% CI for the high FASN expression group. The proteomic data were generated using *n* = 5/group. Western blot data are plotted as fold-change compared to the scramble control for each cell line. Protein expression (from Western blot) was normalized to the Jess system control. Protein data represent n = 2. The Western blot values reported are mean ± SEM. A *t*-test was performed, and asterisks indicate significant differences from the control (* *p* < 0.01, **** *p* < 0.0001). GEPIA, Gene Expression Profiling Interactive Analysis; BLCA, bladder urothelial carcinoma; KD: knockdown; SOX2: transcription factor SOX2; FASN: fatty acid synthase.

Lastly, we utilized shotgun proteomics to gain a better understanding about what pathways are being regulated by SOX2 in UC cells. Proteomics identified a total of 5863 proteins in the As_II control and SOX2-knockdown cells, where 180 were downregulated and 225 proteins were upregulated by 1.5-fold or more (Table S3). Proteomics also confirmed our Western blot expression levels for TRIM29, P63, ALDH3A1, KRT6B, KRT7, and KRT8 following SOX2 KD in As_II cells (reported above). We then performed pathway analysis on the proteins that were significantly increased/decreased greater than 1.5-fold using Reactome (https://reactome.org/, accessed on 1 April 2025) [41]. As shown in Figure 9A and Table 1, SOX2 KD resulted in the downregulation of key enzymes involved in the lipid/cholesterol pathways (activation of gene expression by SREBF (SREBP)). Several proteins involved in the interferon-signaling pathway were also decreased following the KD of SOX2. The unfolded protein response (UPR) and post-translational protein phosphorylation pathways were among the most significant pathways that were upregulated after SOX2 KD (Figure 9B, Table 1). The complete list of Reactome pathways downregulated/upregulated after SOX2 KD are provided in Table S3. Fatty acid synthase (FASN) is a key enzyme of de novo lipogenesis that plays critical roles in cancer cell proliferation and lipid metabolism. Since the lipid/cholesterol pathway was the most significantly downregulated pathway identified after SOX2 KD in As_II cells, we wanted to confirm that SOX2 was regulating FASN levels in all the UC cell lines using Western blot (Figure 9C,D). The results confirm that FASN levels were decreased in all the UC cell lines following SOX2 KD. We next examined the transcript expression of FASN in human bladder urothelial carcinoma (BLCA) tumors vs. normal tissue using GEPIA2 (Gene Expression Profiling Interactive Analysis, http://gepia2.cancer-pku.cn/#index, accessed on 1 April 2025) [42]. Figure 9E demonstrates that FASN transcript expression is higher in bladder tumor tissue than in normal tissue (*p* < 0.01). Additionally, GEPIA2 survival analysis demonstrates that the expression of FASN correlates to a poor overall survival rate in bladder cancer cases (Figure 9F).

## 3. Discussion

The results from our study demonstrate that SOX2 regulates cellular growth rates and clonogenic (colony formation) abilities in UC cells. We observed slower growth rates and fewer colonies in all the UC cell lines after attenuating SOX2 expression using shRNA. These results are on par with a study that determined the importance of the SOX2-IGF-signaling axis on aggressiveness of UC, wherein the authors reported significant decreases in cellular growth rates and an approximately 50% reduction in colonies formed after knockdown of SOX2 with shRNA in 5637 cells [13]. Similarly, a study using a basal (5637) and a luminal (SW780) subtype of UC cells demonstrate that SOX2OT, a long non-coding RNA, regulates SOX2 expression and that knockdown of SOX2OT decreased SOX2 levels and inhibited growth and colony-forming abilities [43].

In Ba/Sq MIUC tumors, P63 is activated and plays an important role in regulating the basal gene program such as the expression of the basal keratins [3]. Similarly, TRIM29 is elevated in Ba/Sq MIUC, and its expression has been used as a sensitive marker to identify UC tumors with SD. P63 has been shown to regulate the expression of TRIM29 and is responsible for driving tumor formation and invasion in Ba/Sq MIUC tumors [26,28,44]. Our results demonstrated that SOX2 KD within the Ba/Sq cells (As_I, As_II, and 5637) decreased the expression of both P63 and TRIM29. To our knowledge, there are no reports of SOX2 directly regulating the expression of TRIM29; however, there are several studies that indicate that P63 is a downstream target of SOX2 in different cancer types and normal epithelial cells [45,46,47]. Therefore, the reduced levels of TRIM29 in our study are likely a result of the SOX2-dependent regulation of P63. In squamous cell carcinomas, it has been shown that SOX2 and P63 co-localize, interact, and co-regulate downstream genes such as ALDH3A1 [48,49]. We observed reduced ALDH3A1 and P63 gene levels after the repression of SOX2; therefore, it is possible that there is a co-regulation of ALDH3A1 by P63 and SOX2 in Ba/Sq UC cells, although this appeared to be UC subtype-dependent in our study. YAP1 signaling has been implicated as a driver of UC stemness, immune evasion, progression, recurrence, and chemoresistance [29,50]. It is known that SOX2 and YAP1 can regulate each other’s expression, and this agrees with the results from our study that showed that YAP1 levels are attenuated in the Ba/Sq UC cells after dampening SOX2 expression with shRNA [51,52,53]. BLIMP1 is an important transcriptional repressor that can regulate SOX2 expression and also regulate the terminal differentiation of epithelial cells and lymphocytes [34,54,55,56]. BLIMP1 negatively regulates cytokine production, and the tumor expression of BLIMP1 can dictate the response to immunotherapies by driving the expression of PD-L1 [57,58,59]. The gene regulating its expression (PRDM1) was found to be enriched in squamous areas of UC tumors; however, the exact role of BLIMP1 in SD is not known [35]. We observed attenuated levels of BLIMP1 following SOX2 KD in the Ba/Sq UC cells and confirmed its expression in squamous areas of As_I tumors.

The Ba/Sq UC subtype is associated with SD and is characterized by the expression of KRT5, KRT6, KRT14, and P63. Our data demonstrate that KD of SOX2 decreases all these Ba/Sq UC markers. SOX2 has been shown to regulate the expression of KRT14 and mark a population of UC stem cells [15]. In a separate study, the ectopic expression of SOX2 was shown to be oncogenic, promote squamous lung tumor development, and drive the expression of SD-associated genes P63, KRT5, and KRT6A [60,61,62]. Also in the lung, it was shown that SOX2 expression influences the tumor immune microenvironment (TIME) and the infiltration of tumor-associated neutrophils (TANs). These alterations to the TIME can influence the squamous state of the tumors and affect their response to chemotherapies [63]. Previously, we demonstrated that knockdown of KRT6 alone was enough to reduce growth, colony formation, basal keratin expression and increase sensitivity to cisplatin in As_I and As_II cells [23]. The current study confirms our previous findings and indicates that SOX2 is an upstream regulator of KRT6 expression that may be regulating the level of SD within UC tumors.

Several immune checkpoint inhibitors (ICIs) are approved for use in advanced UC cases for patients that are ineligible for cisplatin treatment. Still, there is only about a ~30% response rate with their use. The protein level of PD-L1, molecular subtype of tumors, and immune gene expression profile of tumors have all been used as predictors of immunotherapy response, but there is still considerable variability in the results reported from study to study [64,65,66,67,68,69]. Therefore, identifying the molecular determinants of immunotherapy response remains an important objective for developing more effective therapies for UC. FOXA1 is a key marker that helps distinguish the luminal subtype from the Ba/Sq subtype of UC tumors, and SOX2 is known to be a negative upstream regulator of FOXA1 expression in different cancers [70]. This agrees with the results obtained in our study, where we observed FOXA1 induction after depleting SOX2 levels in all four UC cell lines. FOXA1 represses PD-L1 expression and the expression of the interferon response signature of genes, which confers immunotherapy resistance in UC. Recently, it was demonstrated that treatment with interferon (IFN-ɣ) downregulates FOXA1, upregulates PD-L1, and induces the Ba/Sq subtype in MIUC [35,71,72]. These data are in line with our findings, where we observed several of these interferon response proteins downregulated after SOX2 KD (which induced FOXA1). These results suggest that the FOXA1/SOX2 expression ratio may play some role in determining/maintaining either the luminal or Ba/Sq UC subtype. The balance of the expression between these two transcription factors may also be a predictor of the response to ICI or chemotherapy, but this needs further evaluation.

The overexpression of SOX2 in UC cell lines has been shown to impart chemoresistance to cisplatin and gemcitabine treatments [73]. Additionally, SOX2 is known to contribute to the development of cisplatin resistance in other cancers [19,20,21,22]. We confirmed these previous results in the As^3+^-transformed UROtsa cell model (As_I, As_II cells). We observed enhanced sensitivity to cisplatin in both cell lines after SOX2 KD. We also wanted to test another chemotherapy with a different mechanism of action from cisplatin, so we tested its chemosensitivity to pevonedistat (PVD, also known as MLN4924), a small-molecule inhibitor of NEDD8-activating enzyme. Pevonedistat has been shown to inhibit the expression of SOX2 by inactivating the ligase FBXW2 E3, and we have previously demonstrated that PVD can enhance the efficacy of cisplatin in As^3+^-transformed UROtsa cells [11,74]. We also observed increased sensitivity to PVD after SOX2 KD. Taken together, these results indicate that SOX2 contributes to the chemotherapy response in UC.

Fatty acid synthase (FASN) is one of the key enzymes involved in lipogenesis, which is a common metabolic pathway altered in cancer cells. Lipids and cholesterol are needed for the cell membranes of rapidly proliferating cells; they are used as a fuel source for malignant cells, and they can act as second messengers. This pathway is responsible for driving tumor initiation, progression, survival, resistance, and metastatic ability [75,76]. In UC, FASN is differentially expressed in normal vs. tumor tissue, can independently predict muscle invasion, and is associated with cisplatin resistance [75,77]. A recent study identified that simvastatin (HMGCR inhibitor) use can reduce bladder cancer risk, and the FASN inhibitor TVB-2640 is currently being examined in different phase II clinical trials, although it is not currently approved as an independent cancer treatment [78,79,80]. Our proteomics data identified several enzymes involved in lipid/cholesterol metabolism that were repressed after SOX2 KD, and we validated the repression of FASN in all four UC cell lines. Given the role of SOX2 in chemoresistance and its ability to regulate lipid/cholesterol pathways, further consideration should be given to the use of FASN or HMGCR inhibitors as alternative treatments for SOX2+ MIUC tumors that have developed chemoresistance.

This study is not without limitations. The results from the current study should be confirmed in vivo. Specifically, studies evaluating SOX2′s role in SD development in UC tumors would be beneficial. These studies should also be designed to assess the influence of SOX2 on chemo- and immunotherapy responses. Human bioinformatics studies may be designed to answer these same questions in clinical UC cases. The results from our study demonstrated that there were subtype-specific effects after reducing the expression of SOX2. This finding may be important to inform patient stratification or develop a personalized therapy regime for UC patients that have tumors of the luminal or basal subtype and whether they are SOX2- or SOX2+.

In conclusion, this work was performed to build a better understanding of the role SOX2 has in regulating the expression of key genes involved in the Ba/Sq MIUC subtype and in regulating chemotherapy response in MIUC. The results from this study indicate that depleting SOX2 can reduce Ba/Sq markers, increase luminal markers, and enhance the efficacy of cisplatin treatment for MIUC. Additionally, this work identified SOX2-dependent pathways that can be exploited to attenuate disease progression in patients with treatment-resistant MIUC tumors.

## 4. Materials and Methods

### 4.1. Animals

This study adhered to all recommendations dictated in the Guide for the Care and Use of Laboratory Animals of the NIH. The protocol was approved by The University of North Dakota Animal care Committee (IACUC2303-0019). Female SCID mice (NOD.Cg-Prkdc^scid^ Il2rg^tm1Wjl^/SzJ) were used in these studies. The mice were housed five to a cage at 22 °C under a 12 h light/dark cycle. Food and water was available ad libitum. The As^3+^-transformed UROtsa and RT4 cells were injected subcutaneously (SQ) into 5 SCID mice per group. The generation of tumor xenografts using malignant bladder cell lines has been described previously in detail [39,81]. The tumor size was assessed weekly using a ruler, and the animals were sacrificed when the size of the tumor was approximately 1.5–1.8 cm or when dictated by clinical conditions. The animals were euthanized by CO_2_ asphyxiation and conformed to American Veterinary Medical Association Guideline on Euthanasia. Death was confirmed by ascertaining cardiac and respiratory arrest following which the tumors were harvested. Care was taken to ensure that there was no distress to the animals during the procedure.

### 4.2. Cell Culture

The UROtsa cell line has previously been described and authenticated using short tandem repeat (STR) analysis [10,82]. The malignant transformation of the UROtsa cells using arsenite (As^3+^) has also been described [8]. The As^3+^-transformed isolates used in the current study have been previously characterized for their ability to form colonies in soft agar, form spheroids when grown in ultra-low attachment flasks, and form tumors that have focal areas of squamous differentiation when injected subcutaneously in immune-compromised mice [8,10,39,83,84]. The UROtsa cell line was a gift from JR Masters at the Institute of Urology and Nephrology, University College London, UK. The commercial UC cell lines 5637 and RT4 were purchased from American Type Culture Collection (ATCC). Two separate UROtsa As^3+^-transformed cell lines (As_I and As_II), 5637 cells, and RT4 cells were cultured at 37° C and 5% CO_2_ in Dulbecco’s modified Eagle’s medium (DMEM) supplemented with 10% *v*/*v* fetal bovine serum (FBS) and 1% of 100X non-essential amino acids (NEAA, Gibco, Waltham, MA, USA). These UC cells were sub-cultured using TrypLE (Gibco, Waltham, MA, USA) and were fed fresh growth medium every three days.

### 4.3. Lentivirus Transduction

The lentiviral particles used in this study (control and SOX2 shRNA) were sequence 1: pLV[Exp]-U6>hSOX2[shRNA#1]:Terminator-CMV>Luciferase(ns):P2A:Puro (Vector ID: VB231221-1042yah), sequence 2: pLV[Exp]-U6>hSOX2[shRNA#2]:Terminator-CMV>Luciferase(ns):P2A:Puro (Vector ID: VB231221-1044nug), and scramble control pLV[Exp]-U6>Scramble[shRNA#1]:Terminator-CMV>Luciferase(ns):P2A:Puro (Vector ID:VB231221-1036rcq). The lentiviral particles were constructed and packaged by VectorBuilder. The vector ID can be used to retrieve detailed information about the vector on www.vectorbuilder.com, accessed on 1 April 2025. The UROtsa As_I, As_II, 5637, and RT4 cells were seeded in 12-well plates (100,000 cells/well) and infected overnight in medium containing lentiviral particles and polybrene (5 µg/mL). The following morning, the old media was replaced with fresh media for 24 h before selection with puromycin (2 µg/mL). Puromycin selection was for seven days, during which the cells were fed fresh puromycin containing media every three days.

### 4.4. Colony Formation Assay

The colony formation assay has previously been described [23]. Briefly, the UC cells were grown in 6-well plates (200 cells/well). Seeding of control and SOX2-knockdown cells was performed in triplicate on a 6-well plate for each cell line (As_I, As_II, 5637, and RT4). The cells were cultured for 14 days, with the medium changed every 3 days. The resulting colonies were stained with 1 mL of 0.5% crystal violet (Sigma-Aldrich Corp., St. Louis, MO, USA) in 20% methanol for 15 min at room temperature. The plate was washed five times in fresh water, allowed to dry, and colonies were counted using ImageJ (v1.53) software.

### 4.5. RNA Isolation and Real-Time PCR Analysis

The following RNA isolation and real-time PCR analysis has previously been described [11,23]. The cells were washed twice with PBS, then total RNA was isolated from cells using the RNeasy Plus Mini Kit, Qiashredders, and QiaCube instrument following the manufacturer’s protocols (Qiagen, Hilden, Germany). Total RNA (1 µg) was transcribed into cDNA using LunaScript^®^ RT SuperMix Kit (New England Biolabs #E3010L, Ipswich, MA, USA) according to the manufacturer’s protocol. cDNA (2 µL) was combined with 0.2 µM primers and Luna^®^ Universal qPCR Master Mix (New England Biolabs #M3003E) according to the manufacturer’s protocol in a total volume of 20 µL. qPCR gene amplification was monitored by SYBR Green fluorescence using BioRad CFX96 Touch Real-Time PCR Detection System (Bio Rad Laboratories, Hercules, CA, USA). qPCR cycle conditions were 1 cycle of 2 min at 95 °C, and 40 cycles of 5 s at 95 °C and 30 s at the annealing temperature of 60 °C. Expression levels were determined from the threshold cycle (Ct) values using the method of 2^−∆∆Ct^ using 18S as the reference control gene. Primers were obtained from Integrated DNA Technology (IDT, Coralville, IA, USA) and are listed in Table S1.

### 4.6. Simple Western Blot Analysis

The use of Simple Western blot to measure protein expression has been previously described [11,23,85,86]. Briefly, the cells were washed twice with PBS then dissolved in 1X ( Radio-immunoassay Precipitation Assay (RIPA) lysis buffer supplemented with PMSF, protease inhibitor cocktail, and sodium orthovandate (Santa Cruz Biotechnology, Dallas, TX, USA). The cell suspension was sonicated and the lysate was centrifuged to remove cellular debris. Protein lysates were quantified using the Pierce BCA protein assay kit (Thermo-Scientific Pierce, Rockford, IL, USA). Diluted protein lysates were combined with 5X fluorescent master mix (ProteinSimple, San Jose, CA, USA), which contains reducing agent (dithiothreitol), fluorescent standards, and a system control protein (26 kDa). Denatured (95 °C for 5 min) lysates were separated and the immunodetection of target proteins was performed using a capillary-based Jess Simple Western instrument (ProteinSimple, San Jose, CA, USA) according to the manufacturer’s protocol. The ProteinSimple system control protein (26 kDa) served as an internal control and was also used to normalize protein expression. Duplicates of each sample (4 µL) were analyzed for target protein expression. The protein lysate concentrations used to detect each target protein and antibodies/dilutions are listed in Table S2. The uncropped blots are provided in Figures S2, S4, S6, and S7.

### 4.7. Immunohistochemistry

Subcutaneous tumor tissue obtained from mice injected with As^3+^-transformed UROtsa or RT4 cells was used in this study. Serial sections of FFPE tumor tissue were cut at 3–5 µm. Immunohistochemistry (IHC) was performed using a Leica Bond RX stainer (Leica Biosystems, Deer Park, IL, USA). The tissue slides were stained using the manufacturers solutions and protocol. Briefly, the slides are dewaxed (Bond Dewax, Leica Biosystems, Deer Park, IL, USA) before undergoing antigen retrieval (Epitope Retrieval 1, Citrate, Leica Biosystems, Deer Park, IL, USA). The antigen retrieval was for 20 min. Slides were then incubated in Peroxide Block (Leica Biosystems, Deer Park, IL, USA) for 5 min before incubating with the primary antibody for 30 min. The primary antibodies used in this study along with their dilutions are listed in Table S2. The detection system used was the Refine Detection System (Leica Biosystems, Deer Park, IL, USA) which includes the secondary antibody polymer, 3,3-diaminobenzidine (DAB), and Hematoxylin. When the run was complete, the slides were removed from the stainer and were rinsed in distilled water, dehydrated in graded ethanol, cleared in xylene, and cover-slipped.

### 4.8. Luciferase Assay for Growth/Viability

The luciferase assay for growth and viability has previously been described [23]. Briefly, the control and SOX2-knockdown cells were seeded at various densities in a 96-well plate (156–20,000 cells/well) to demonstrate the correlation between luminescence intensity and cell number for the UC cell lines. For growth, all cells were seeded at the same density (2500 cells/well). The cells were then cultured for 24 h, and luminescence was read on a Tecan Spark plate reader 10 min after the addition of sterile filtered luciferin (Revvity Health Sciences Inc. (Waltham, MA, USA, Cat. # 122799), 150 µg/mL). After luminescence was read, the luciferin-containing media was replaced with fresh media (no luciferin), and the cells were placed back in the incubator. Luminescence readings were then obtained daily for six days (24–144 h) after seeding. For cell viability after cisplatin and pevonedistat treatments, As_I and As_II cells were seeded at 2500 cells/well. The cells were then cultured for 24 h and then treated with various doses of cisplatin or pevonedistat-containing media. The treatment lasted 72 h before luminescence was measured. There were 8 replicates for each dose.

### 4.9. Proteomics Analysis

Total protein from each sample (n = 5/group) was reduced, alkylated, and purified by chloroform/methanol extraction prior to digestion with sequencing-grade modified porcine trypsin (Promega Corporation, Madison, WI, USA). Tryptic peptides were then separated by reverse phase XSelect CSH C18 2.5 um resin (Waters Corporation, Milford, MA, USA) on an in-line 150 × 0.075 mm column using an UltiMate 3000 RSLCnano system (Thermo Fisher Scientific, Waltham, MA, USA). Peptides were eluted using a 60 min gradient from 98:2 to 65:35 buffer A:B ratio. Eluted peptides were ionized by electrospray (2.2 kV) followed by mass spectrometric analysis on an Orbitrap Exploris 480 mass spectrometer (Thermo Fisher Scientific, Waltham, MA, USA).

For wide-window acquisitions, the Orbitrap Exploris was configured to acquire a precursor scan (385–1015 *m*/*z*, 60,000 resolution, normalized AGC target 100%, maximum injection time 50 ms) followed by 50x 12 *m*/*z* DIA spectra (12 *m*/*z* precursor isolation windows at 15,000 resolution, normalized AGC target 100%, maximum injection time 33 ms) using a staggered window pattern with optimized window placements. Precursor spectra were acquired after each DIA duty cycle. Buffer A was 0.1% formic acid with 0.5% acetonitrile and buffer B was 0.1% formic acid with 99.9% acetonitrile. Following data acquisition, data were searched using Spectronaut (Biognosys version 18.7) against the UniProt Homo sapiens database (June 2024) using the directDIA method with an identification precursor and protein q-value cutoff of 1%, generate decoys set to true, protein inference workflow set to maxLFQ, inference algorithm set to IDPicker, quantity level set to MS2, cross-run normalization set to false, and protein grouping quantification set to median peptide and precursor quantity. Protein MS2 intensity values were assessed for quality using ProteiNorm [87]. The data were normalized using vsn and analyzed using proteoDA to perform statistical analysis using Linear Models for Microarray Data (limma) with empirical Bayes (eBayes) smoothing to the standard errors [88,89]. Proteins with an FDR-adjusted *p*-value < 0.05 and a fold change > 1.5 were considered significant. Pathway analysis was generated using Reactome (https://reactome.org/, accessed on 1 April 2025) and the differentially expressed proteins (increased or decreased) as the input [41]. A fold change greater than 1.5-fold and an FDR-adjusted *p*-value < 0.05 was used for pathway analysis.

### 4.10. Statistical Analysis

Unless otherwise stated, experiments were performed in either duplicate or triplicate, and the results are expressed as the mean ± SEM. Statistical analyses were performed using GraphPad Prism^®^ software version 10.3.1 using *t*-test. The level of significance was *p* < 0.05.

## Figures and Tables

**Table 1:** Most significantly altered pathways after SOX2 knockdown in UROtsa As_II cells.

Downregulated Pathways	Protein	Fold (SOX2 KD/Con)	*p*-Value (FDR Adjusted)
Activation of gene expression by SREBF (SREBP)	HMGCS1	0.67	3.88 × 10^−6^
	FASN	0.60	2.34 × 10^−7^
	MVK	0.60	1.50 × 10^−3^
	HMGCR	0.59	9.23 × 10^−6^
	SP1	0.59	3.18 × 10^−5^
	TM7SF2	0.57	1.00 × 10^−3^
	SQLE	0.54	4.14 × 10^−8^
	DHCR7	0.53	1.36 × 10^−8^
	FDFT1	0.52	4.60 × 10^−9^
	SCD	0.43	5.52 × 10^−8^
Interferon Signaling	OAS1	0.67	2.05 × 10^−5^
	TUBB2A	0.66	2.86 × 10^−5^
	TRIM14	0.65	2.50 × 10^−4^
	HSPA2	0.65	3.12 × 10^−6^
	MX2	0.65	1.05 × 10^−3^
	TUBB4B	0.65	5.37 × 10^−6^
	HSPA1A	0.65	1.10 × 10^−7^
	IFIT5	0.65	4.14 × 10^−3^
	NUP153	0.64	2.47 × 10^−6^
	IFIT2	0.64	9.91 × 10^−4^
	EIF4A2	0.63	2.25 × 10^−5^
	IFIT3	0.59	1.34 × 10^−5^
	STAT1	0.58	7.77 × 10^−8^
	TUBA1C	0.57	3.48 × 10^−2^
	IFIT1	0.54	1.51 × 10^−7^
	HERC5	0.50	1.93 × 10^−5^
	ISG15	0.49	1.75 × 10^−8^
	IFITM1	0.45	7.94 × 10^−7^
	MX1	0.35	1.87 × 10^−9^
Upregulated Pathways			
Unfolded Protein Response (UPR)	DNAJC3	1.94	7.19 × 10^−9^
	HYOU1	1.77	2.31 × 10^−9^
	FKBP14	1.70	2.65 × 10^−6^
	HSP90B1	1.65	4.41 × 10^−9^
	HDGF	1.62	8.96 × 10^−8^
	HSPA5	1.59	4.72 × 10^−9^
	MYDGF	1.53	2.12 × 10^−5^
Post-translational protein phosphorylation	LGALS1	6.52	6.55 × 10^−10^
	C3	3.19	1.03 × 10^−7^
	LAMB1	2.62	2.42 × 10^−11^
	MELTF	2.13	3.84 × 10^−8^
	CKAP4	2.01	2.42 × 10^−11^
	CCN1	1.95	1.84 × 10^−3^
	CDH2	1.95	1.23 × 10^−9^
	DNAJC3	1.94	7.19 × 10^−9^
	CSF1	1.78	1.64 × 10^−3^
	HSP90B1	1.65	4.41 × 10^−9^
	P4HB	1.60	2.40 × 10^−8^
	CALU	1.57	5.40 × 10^−9^

Quantitative proteomics were performed on n = 5 for each group (control and SOX2 KD). Pathways were identified using Reactome (https://reactome.org/, accessed on 1 April 2025). Green fold values indicate downregulation and red fold values indicate upregulation. KD: knockdown; SOX2: transcription factor SOX2.

## Data Availability

The original contributions presented in this study are included in the article/Supplementary Material. Further inquiries can be directed to the corresponding author.

## Supplementary Materials

The following supporting information can be downloaded at: https://www.mdpi.com/article/10.3390/cells14130949/s1, Figure S1: SOX2 protein expression after lentiviral knockdown in UC cells; Figure S2: Uncropped blots for Figure 1; Figure S3: Signal stability, sensitivity, and dynamic range of luminescence assay; Figure S4: Uncropped blots for Figure 4; Figure S5: Gene expression; Figure S6: Uncropped blots for Figure 6; Figure S7: Uncropped blots for Figure 7; Figure S8: Uncropped blot for Figure 9; Table S1: List of primers used in the study; Table S2: Antibodies used for Western analysis and Immunohistochemistry (IHC); Table S3: Quantitative proteomic data and Reactome pathway analysis.
