# Supplementary material for: SOX2 Regulates Growth, Expression of Basal/Luminal Markers, and Chemotherapy Response in Urothelial Carcinoma
Source: Cells. 2025 Jun 20;14(13):949. doi: 10.3390/cells14130949 (PMC12249187; doi:10.3390/cells14130949)
Supplement: Supplementary file 1 [file cells-14-00949-s001.zip › Supplementary/Revised Supplementary Combined.pdf]

Figure S1. SOX2 protein expression after lentiviral knockdown in UC cells.

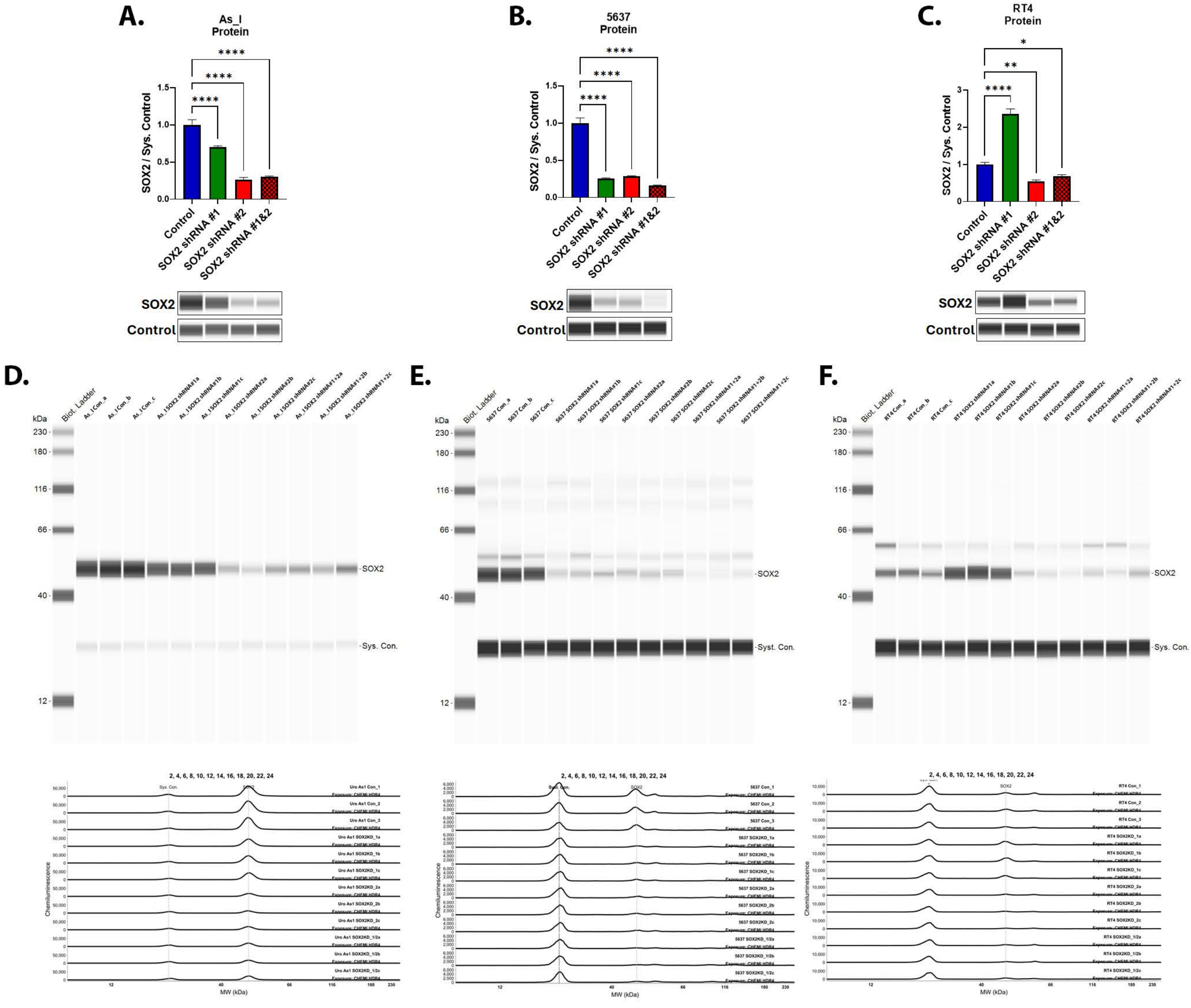

Figure S2. Uncropped blots for Figure 1

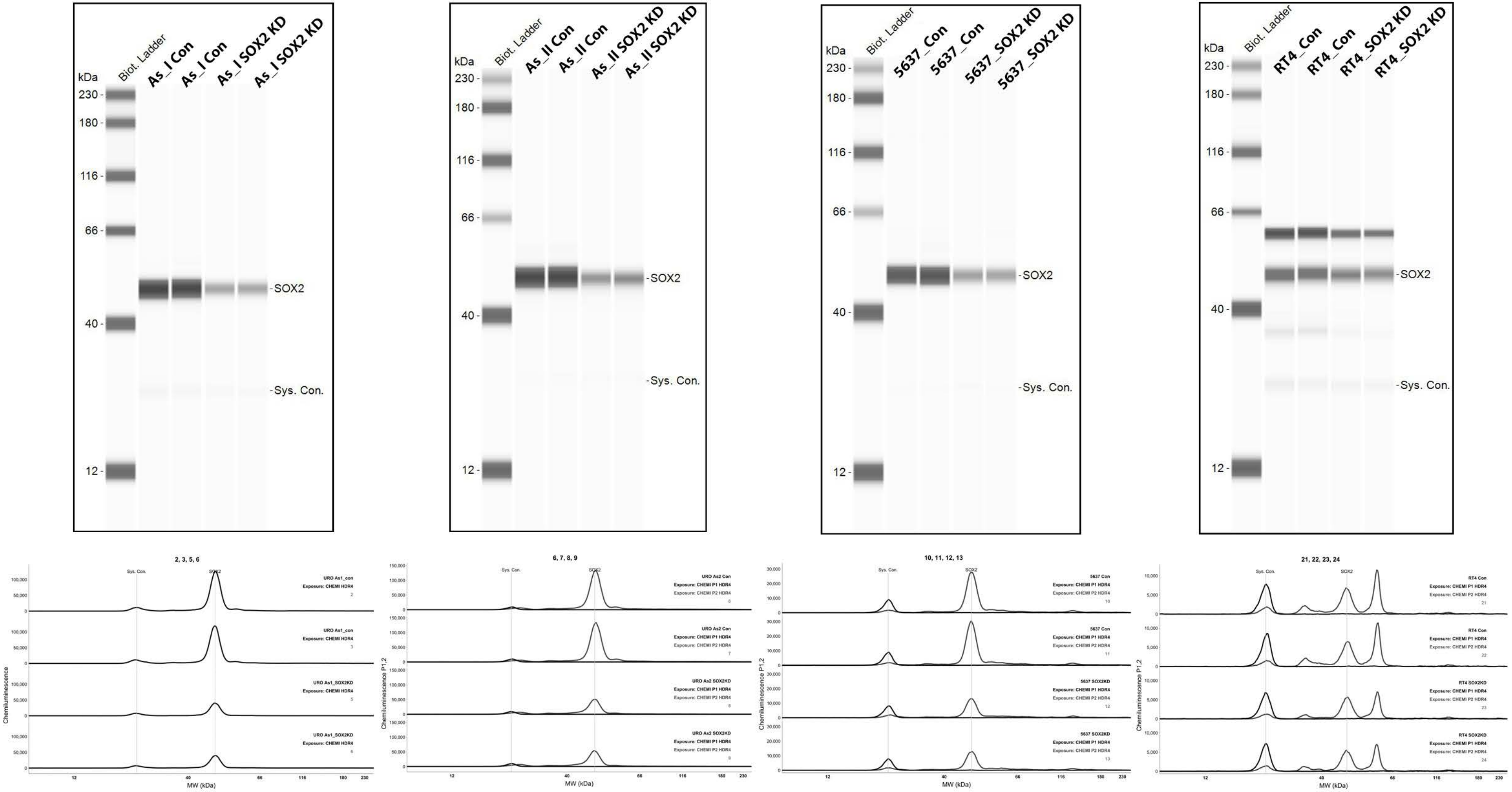

Figure S3. Signal stability, sensitivity, and dynamic range of luminescence assay

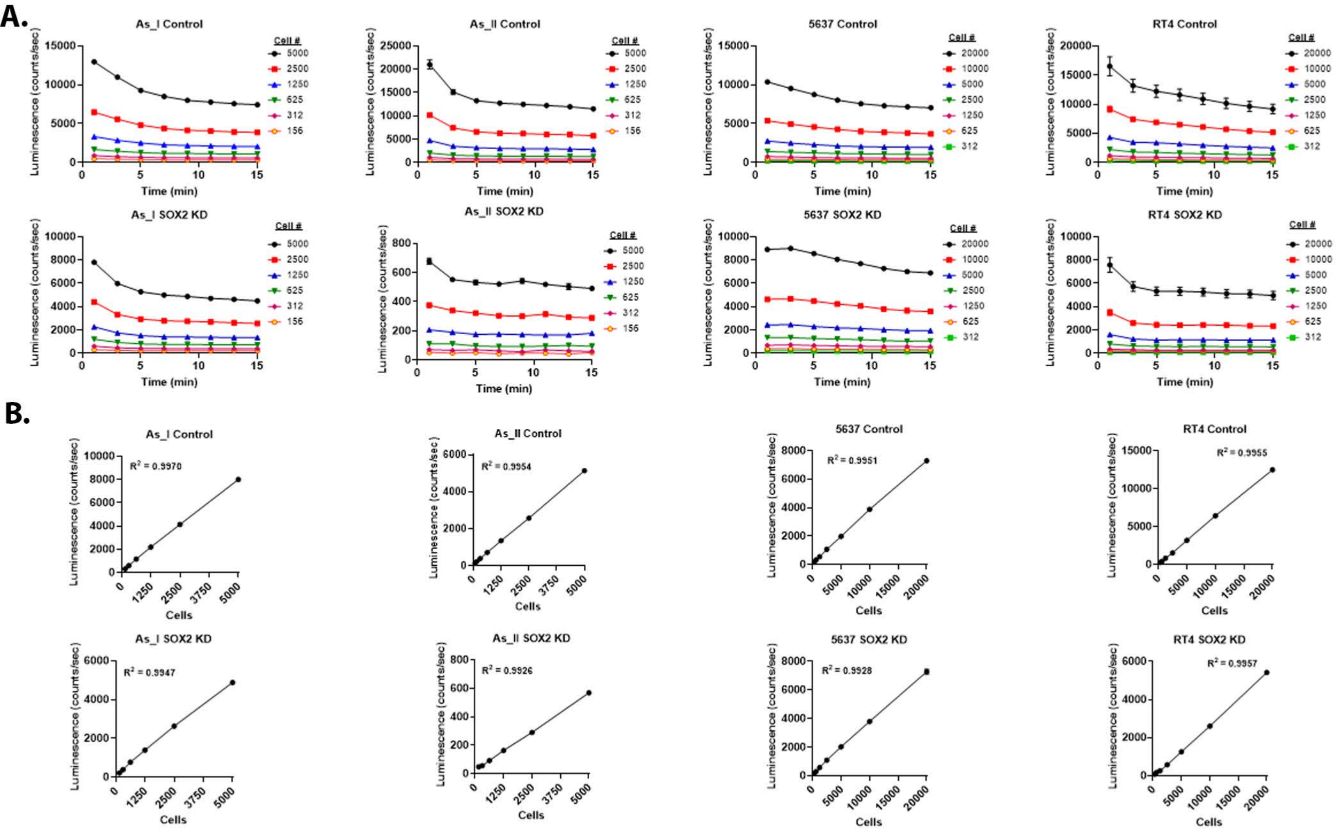

Figure S4. Uncropped blots for Figure 4

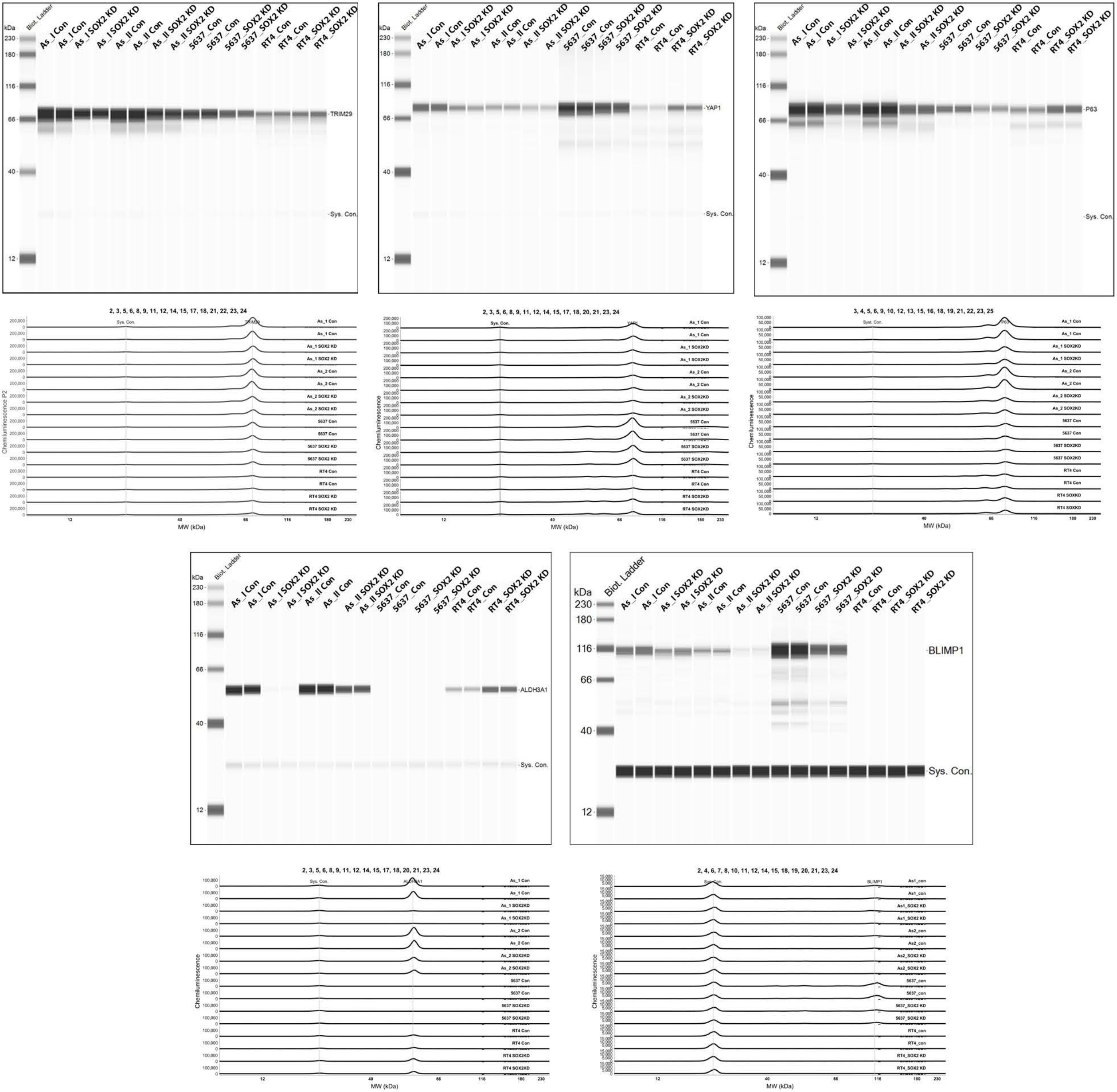

Figure S5. Gene expression

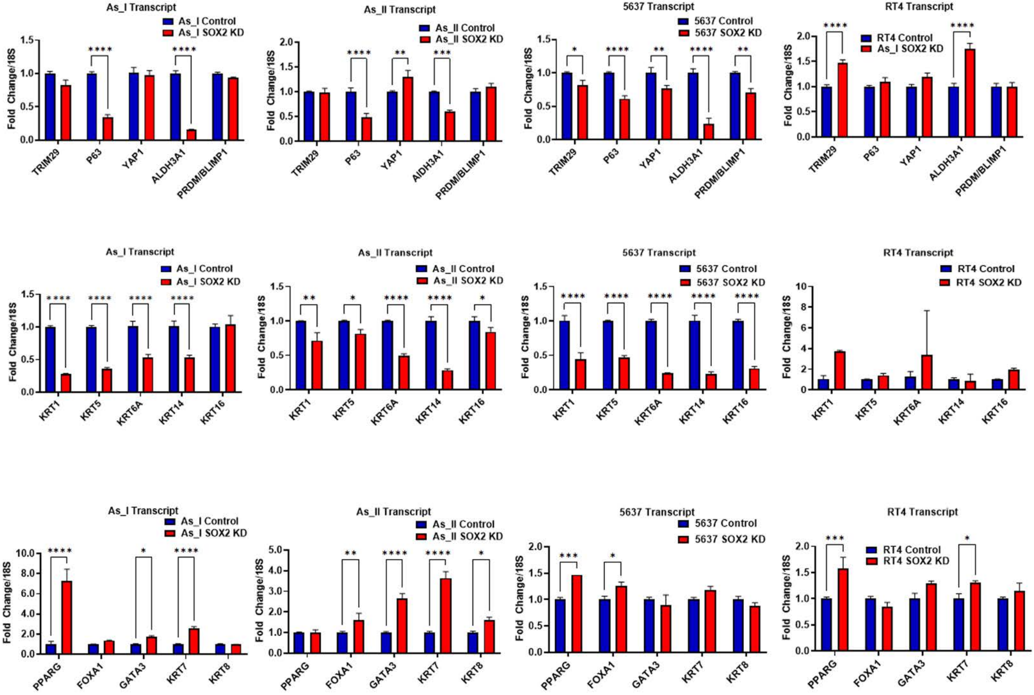

Figure S6. Uncropped blots for Figure 6

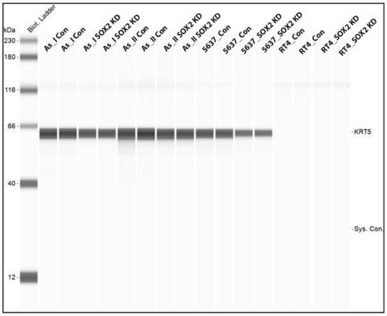

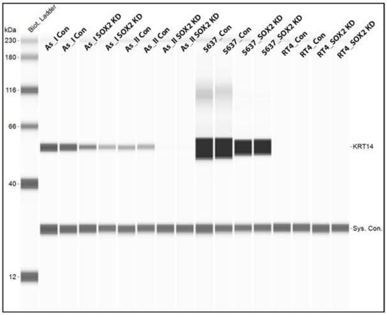

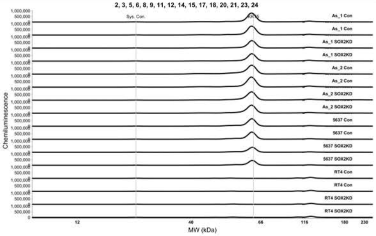

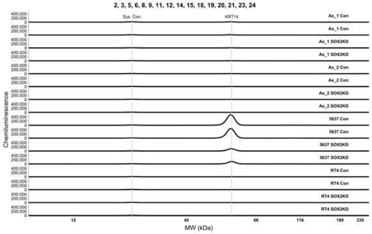

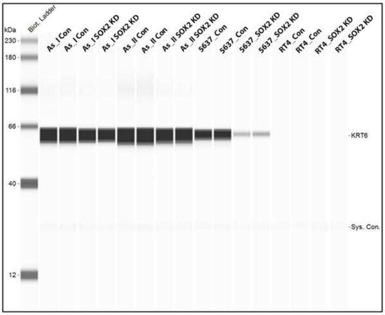

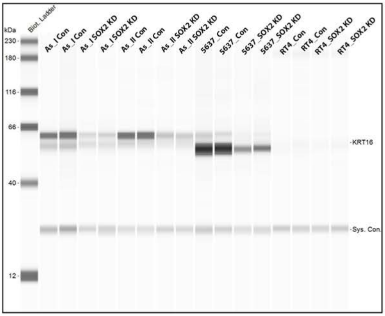

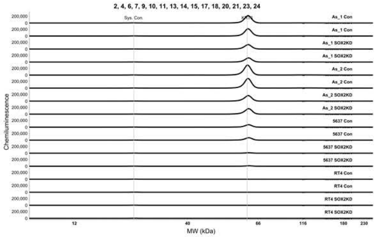

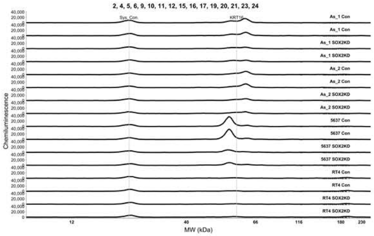

Figure S7. Uncropped blots for Figure 7

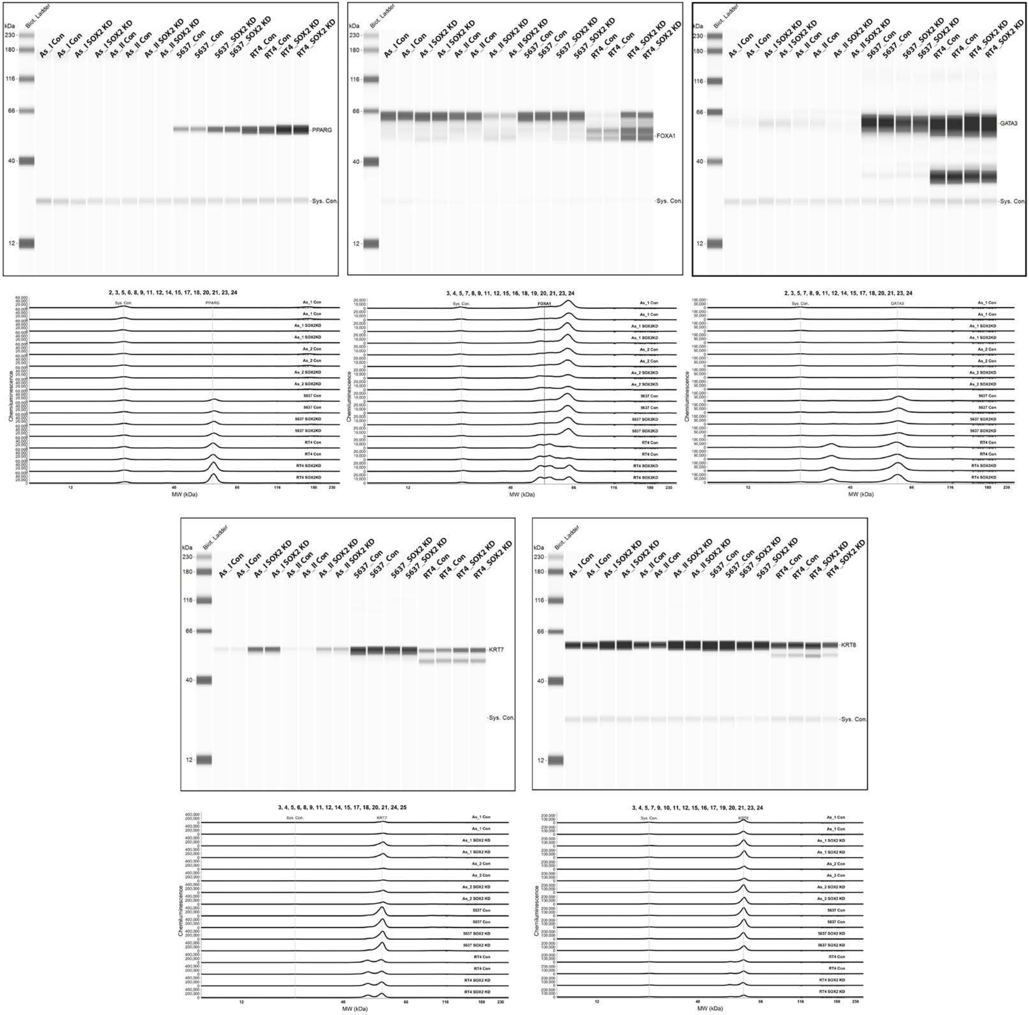

Figure S8. Uncropped blot for Figure 9

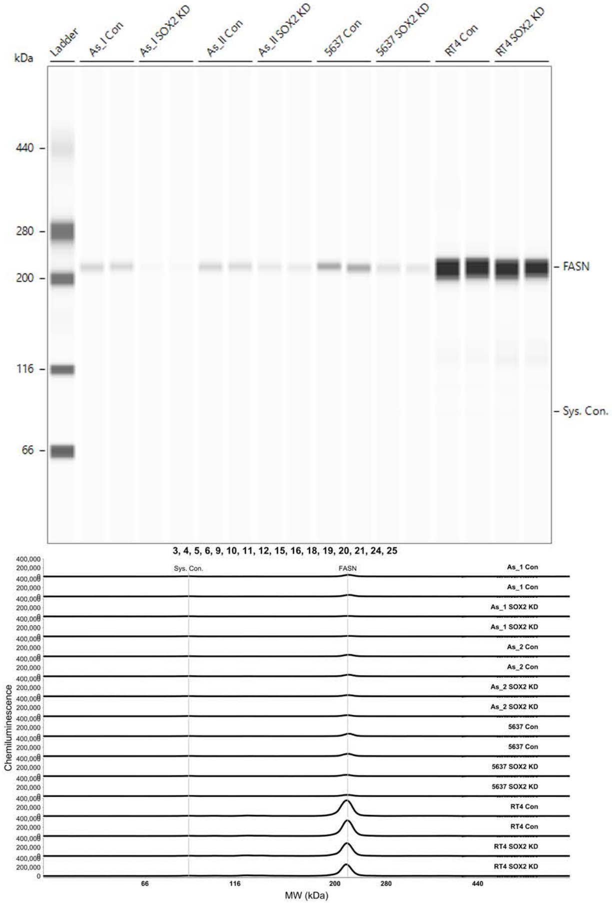

Table S1. List of primers used in the study

Genes	Product	Catalog No./<br>unique Assay ID	Source
KRT1	PrimeTime qPCR primers	Hs.PT.58.24741966	Integrated DNA Technologies
KRT5	PrimeTime qPCR primers	Hs.PT.58.14446018	Integrated DNA Technologies
KRT6A	PrimeTime qPCR primers	Hs.PT.58.26132549.g	Integrated DNA Technologies
KRT14	PrimeTime qPCR primers	Hs.PT.58.4592110	Integrated DNA Technologies
KRT16	PrimeTime qPCR primers	Hs.PT.58.40837518.g	Integrated DNA Technologies
TP63	PrimeTime qPCR primers	Hs.PT.58.2966111	Integrated DNA Technologies
YAP1	PrimeTime qPCR primers	Hs.PT.58.14881945	Integrated DNA Technologies
PPARG	PrimeTime qPCR primers	Hs.PT.58.25464465	Integrated DNA Technologies
FOXA1	PrimeTime qPCR primers	Hs.PT.58.1788586	Integrated DNA Technologies
GATA3	PrimeTime qPCR primers	Hs.PT.58.4308511	Integrated DNA Technologies
KRT7	PrimeTime qPCR primers	Hs.PT.58.3835469	Integrated DNA Technologies
KRT8	PrimeTime qPCR primers	Hs.PT.58.22681010	Integrated DNA Technologies
ALDH3A1	PrimeTime qPCR primers	Hs.PT.56a.24823646.g	Integrated DNA Technologies
TRIM29	PrimeTime qPCR primers	Hs.PT.58.38937263	Integrated DNA Technologies
PRDM1/<br>BLIMP1	PrimeTime qPCR primers	Hs.PT.56a.39313533.g	Integrated DNA Technologies

*18S rRNA primers were custom ordered from IDT- Upper: CGCCGCTAGAGGTGAAATTC
Lower: TTGGCAAATGCTTTCGCTC

Table S2. Antibodies used for Western analysis and Immunohistochemistry (IHC)

Antigen	Source	Cat. No	Protein lysate conc. (μg/μL)<br>For Western blot	Dilution For Western	Dilution For IHC
SOX2	Cell Signaling	3579	0.50	1:100	
KRT5	Invitrogen	PA5-29670	0.03	1:1600	
KRT6	Santa Cruz	sc-514520	0.03	1:1600	
KRT14	Invitrogen	PA5-16722	0.06	1:200	
KRT16	Abcam	ab76416	0.50	1:100	
ALDH3A1	Novus	NBP2-47551	0.25	1:200	1:200
TRIM29	Abcam	ab244380	0.03	1:200	
YAP1	Proteintech	13584-1-AP	0.13	1:400	1:2000
P63	Abcam	ab124762	0.03	1:200	
PPARG	Cell Signaling	2435	0.50	1:200	
FOXA1	Santa Cruz	SC-101058	0.50	1:50	
GATA3	Cell Signaling	5852	0.50	1:50	
KRT7	Invitrogen	MA5-11986	0.06	1:800	
KRT8	Abcam	ab53280	0.06	1:400	
BLIMP1	Cell Signaling	9115	0.50	1:100	1:100

Supporting information legends

Figure S1. SOX2 protein expression after lentiviral knockdown in UC cells. Scramble (control) shRNA or different shRNA sequences targeting human SOX2 (sequence 1, sequence 2, or combination of sequence 1 + sequence 2) were evaluated for ability to reduce SOX2 expression relative to the scramble control. (A) Quantification of SOX2 protein expression measured by Western blot after lentiviral knockdown of SOX2 in UROtsa As_I cells. (B) Quantification of SOX2 protein expression measured by Western blot after lentiviral knockdown of SOX2 in 5637 cells. (C) Quantification of SOX2 protein expression measured by Western blot after lentiviral knockdown of SOX2 in RT4 cells. (D-F) Uncropped Western blot images with corresponding electropherograms showing all biological (n=3) replicates. Quantification data represented as mean ± SEM and plotted as fold-change from control. An ordinary one-way ANOVA was performed followed by a Dunnett's multiple comparisons test to demonstrate significance. Asterisks indicate significant differences from control values (*$p < 0.05$, ** $p < 0.01$,****$p<0.0001$).

Figure S2. Uncropped Western blots for Figure 1. Displays all uncropped Western blot images with corresponding electropherograms used to generate Figure 1.

Figure S3. Signal Stability, Sensitivity, and Dynamic Range of Luminescence Assay. (A) Luminescence intensity was read every 2 minutes for a duration of 15 minutes demonstrating that signal was stabilized by 10 minutes in all control and SOX2 KD UC cells. (B) Luminescence intensity of two-fold serially diluted control and SOX2 knockdown UC cells. R^2 shows the linear correlation between luminescence intensity and cell number. There were 8

replicates for each dilution on a 96-well plate and the mean intensity of the replicates was plotted.

Figure S4. Uncropped Western blots for Figure 4. Displays all uncropped Western blot images with corresponding electropherograms used to generate Figure 4.

Figure S5. Gene expression from control and SOX2 knockdown UC cells. Gene expression from As_I, As_II, 5637, and RT4 cells. The scramble control (blue bars) and SOX2 KD (red bars) expression are shown. All data is plotted as fold-change compared to the scramble control. Gene expression was normalized to the 18S housekeeping gene. The gene measurements were performed in triplicates and the values reported are mean ± SEM. A t test was performed, and asterisks indicate significant differences from the control (* $p < 0.05$, ** $p \leq 0.01$, *** $p \leq 0.001$, **** $p \leq 0.0001$).

Figure S6. Uncropped Western blots for Figure 6. Displays all uncropped Western blot images with corresponding electropherograms used to generate Figure 6.

Figure S7. Uncropped Western blots for Figure 7. Displays all uncropped Western blot images with corresponding electropherograms used to generate Figure 7.

Figure S8. Uncropped Western blots for Figure 9. Displays all uncropped Western blot images with corresponding electropherograms used to generate Figure 9.

Table S1. List of primers used in the study

Table S2. Antibodies used for Western and immunohistochemistry analysis

Table S3. Quantitative proteomic data and Reactome pathway analysis
